# Single-Cell Transcriptome Analysis Identifies Ligand–Receptor Pairs Associated With BCP-ALL Prognosis

**DOI:** 10.3389/fonc.2021.639013

**Published:** 2021-03-10

**Authors:** Liang Wu, Minghao Jiang, Ping Yu, Jianfeng Li, Wen Ouyang, Chong Feng, Wei Li Zhao, Yuting Dai, Jinyan Huang

**Affiliations:** ^1^Shanghai Institute of Hematology, State Key Laboratory of Medical Genomics, National Research Center for Translational Medicine, Shanghai Rui Jin Hospital, Shanghai Jiao Tong University School of Medicine, Shanghai, China; ^2^School of Life Sciences and Biotechnology, Shanghai Jiao Tong University, Shanghai, China

**Keywords:** BCP-ALL, scRNA-seq, ligand–receptor pairs, machine learning, prognosis

## Abstract

B cell precursor acute lymphoblastic leukemia (BCP-ALL) is a blood cancer that originates from the abnormal proliferation of B-lymphoid progenitors. Cell population components and cell–cell interaction in the bone marrow microenvironment are significant factors for progression, relapse, and therapy resistance of BCP-ALL. In this study, we identified specifically expressed genes in B cells and myeloid cells by analyzing single-cell RNA sequencing data for seven BCP-ALL samples and four healthy samples obtained from a public database. Integrating 1356 bulk RNA sequencing samples from a public database and our previous study, we found a total of 57 significant ligand–receptor pairs (24 upregulated and 33 downregulated) in the autocrine crosstalk network of B cells. Via assessment of the communication between B cells and myeloid cells, another 29 ligand–receptor pairs were discovered, some of which notably affected survival outcomes. A score-based model was constructed with least absolute shrinkage and selection operator (LASSO) using these ligand–receptor pairs. Patients with higher scores had poorer prognoses. This model can be applied to create predictions for both pediatric and adult BCP-ALL patients.

## Introduction

B cell precursor acute lymphoblastic leukemia (BCP-ALL) is a hematological malignant neoplasm caused by the abnormal proliferation and accumulation of B-lymphoblastic progenitor cells in the bone marrow (1). Although the 5-year survival rate of pediatric BCP-ALL has surpassed 90% in some developed countries, it remains a main factor in cancer-related death in children and has high morbidity (2, 3). Chemotherapy and targeted therapy are effective treatments for the majority of incipient BCP-ALL patients. However, about 15–20% of such patients will relapse within 5 years, become drug resistant, and eventually die (4). This is in part due to the high heterogeneity of BCP-ALL and to extensive remodeling of the immune microenvironment (5).

Bulk RNA sequencing (RNA-seq) is widely used to analyze the transcriptomic landscape of BCP-ALL. It can reflect the average expression level of various cell types in bone marrow or peripheral blood as a whole. However, our knowledge of the microenvironment of leukemia cells is limited to only bulk RNA-seq data. As single-cell RNA sequencing (scRNA-seq) technology in cancer research becomes increasingly promoted and applied, it has come to provide insights into the analysis of the complexity of cellular composition as well as the heterogeneity of the tumor microenvironment (TME) (6, 7). The use of scRNA-seq can help us gain a deep understanding of the pathogenesis of BCP-ALL (5).

TME plays a crucial role in tumorigenesis and tumor progression, drug tolerance, and immune infiltration (8). The process of tumor development is inhibited by immune cells, and conversely, tumor cells secrete immunoregulatory factors and constantly reshape the microenvironment, leading to a change in the microenvironment in favor of tumor growth and invasions (9–12).

The communication among various cells in TME is mainly mediated through ligand–receptor interactions either in soluble or membrane bound form (13). Checkpoint inhibitors that operate based on the ligand–receptor interaction have become powerful tools for clinical therapy (14). In recent years, several studies have been conducted on the cell–cell crosstalk of TME based on scRNA-seq. For example, Kumar et al. characterized cell–cell communication across all cell types in the microenvironment of mouse tumor models, including melanoma, breast cancer, and lung cancer, and found that the expression of individual ligand–receptor pairs was closely linked to tumor growth rate (15). By analyzing single-cell data in glioma, Shi et al. found that cellular interactions between glioma stem cells and tumor-associated macrophages could affect the prognosis of glioma patients (16). These works provide the references and analytical workflow for cell–cell communications.

However, current research on cell–cell communication focuses on solid tumors. Our understanding of intercellular interactions in leukemia, such as BCP-ALL, remains limited. Previous research has found the extensive remodeling of the TME in BCP-ALL, and a non-classic mononuclear subpopulation is enriched within the myeloid compartment. This subpopulation has prognostic implications for BCP-ALL (5). How myeloid cells affect tumorigenesis and the communication between myeloid and neoplastic B cells in the BCP-ALL TME has not been fully explored. To investigate cell–cell communication in BCP-ALL in depth, we analyzed scRNA-seq data of seven BCP-ALL samples and four healthy samples. Among the seven BCP-ALL samples, five of them are *ETV6*-*RUNX1* fusion. They belong to low-risk subtype and occurs mostly in children. Two of them are *BCR*-*ABL1* fusion (also called Ph+), which belong to high-risk subtype (17, 18). Totally 57 ligand–receptor pairs were found in the autocrine crosstalk network of tumor-related B cells, and 29 were detected in the paracrine crosstalk network between B cells and myeloid cells. A robust least absolute shrinkage and selection operator (LASSO) regression model was constructed using ligand–receptor pairs to predict prognoses for both pediatric and adult BCP-ALL patients.

## Materials and Methods

### Datasets

The scRNA-seq data related to BCP-ALL in recent five years was searched from Gene Expression Omnibus (GEO, https://www.ncbi.nlm.nih.gov/geo/) and only the dataset GSE134759 was found. Bulk RNA-seq and clinical data of BCP-ALL used for survival analysis and prognostic model construction was downloaded from the Therapeutically Applicable Research to Generate Effective Treatments (TARGET, https://ocg.cancer.gov/programs/target). The TARGET ALL P2 cohort with 532 samples was obtained by R package TGCAbiolinks (v2.16.3). And 133 primary diagnosis BCP-ALL samples whose definition was primary blood derived cancer (bone marrow) were used in the downstream analysis. Another bulk RNA-seq and the clinical dataset was collected from five significant patient cohorts (19–26), including 1,223 BCP-ALL cases available from our previous study (17). This dataset was used for Spearman’s correlation calculation and prognostic model validation. The 36 tumor cohorts of The Cancer Genome Atlas (TCGA) used for validating the model were downloaded *via* R package TGCAbiolinks (v2.16.3). Ligand–receptor pairs were collected from several public databases (13, 27).

### scRNA-seq Data Analysis

All steps for scRNA-seq data processing and cell–cell communication analysis as well as for the machine learning model development described below were performed with R (v4.0.1). For the seven BCP-ALL and four healthy samples, cells for which less than 500 genes or over 10% genes derived from the mitochondrial genome were first filtered out. To remove doublets, cells with more than 5,000 genes were also filtered. All of the 11 samples were preprocessed and normalized using SCTransform, with default parameters implemented in Seurat (v3.5.1) package individually (28, 29). Seurat anchor-based integration method was used to correct the batch and merge multiple samples (30). Cell-type annotation was performed by R package cellassign (v0.99.21) in conjunction with manual comparison of the expression of marker genes among different clusters (31). The pheatmap (v1.0.12) was used to plot heatmap for cell-type annotation using 5,000 randomly selected cells. This was only done to plot the heatmap. The inferCNV (v1.4.0) was used to calculate the copy number variation (CNV) levels of tumor samples.

### Cell–Cell Communication Analysis

The differential expression of genes between the BCP-ALL samples and healthy samples separately for B cells and myeloid cells was compared using MAST (v1.14.0) (32). Significant genes with adjusted P-value < 0.05 were mapped to ligand–receptor pair databases. To further investigate the correlations in the ligand–receptor pairs, Spearman’s correlation coefficient was calculated to check the co-expression level of individual pairs. Any pair with an adjusted P-value < 0.05 and coefficient > 0.3 was considered to be significant. Gene set enrichment analysis (GSEA) was performed using fgsea (v1.14.0). Pathway enrichment analysis was performed using clusterProfiler (v3.16.1) (33).

### Survival Analysis

Kaplan-Meier and log-rank tests were performed using the survival (v3.2-3) and survminer (v0.4.8) packages to construct and compare survival curves for the LASSO prediction model or specific genes. For specific genes, the patients were divided into high- or low-expression groups according to the mean expression of this gene, and P-value < 0.05 was considered to denote significance.

### Machine Learning Model Development

The LASSO regression model implemented in the glmnet (v4.0-2) package was fitted to predict the patient prognosis based on ligand–receptor pairs between B cells and myeloid cells. LASSO regression penalizes the data-fitting standard by eliminating predictive variables with less information to generate simpler and more interpretable models. To evaluate the variability and reproducibility of the estimates produced by the LASSO Cox regression model, we repeated the regression fitting process for each of the 1,000 leave-10%-out cross-validation evaluations. Genes with non-zero coefficient estimates were retained across all 1,000 evaluations. For each of these genes, the final model coefficient was taken as the average of the coefficient estimates obtained for the set of cross-validation evaluations. The recursive partitioning survival model available in the rpart (v4.1-15) package was used to dichotomize patients into low- and high-score groups. Multivariable Cox-proportional hazard model was used to check the independent prognostic effect. The risk group was defined by our previous study (17). In pediatric BCP-ALL, patients with *TCF3-PBX1*, *ETV6-RUNX1*/-like, *DUX4* fusions, *ZNF384*/*ZNF362* fusions, and high hyperdiploidy (51–65/67 chromosomes) were defined as low-risk. Patients with hyperdiploidy (≤50 chromosomes), *PAX5* and *CRLF2* fusions were defined as intermediate-risk. While patients with *MEF2D* fusions, *BCR-ABL1*/Ph-like, and KMT2A fusions were defines as high-risk. And in adult BCP-ALL, patients with *DUX4* fusions, *ZNF384*/*ZNF362* fusions, and hyperdiploidy were defined as intermediate-risk, and patients with *MEF2D* fusions, *TCF3-PBX1*, *BCR-ABL1*/Ph-like, and *KMT2A* fusions were defined as high-risk (17).

## Results

### Cellular Heterogeneity Within the Immune Microenvironment of BCP-ALL

**Figure 1:**
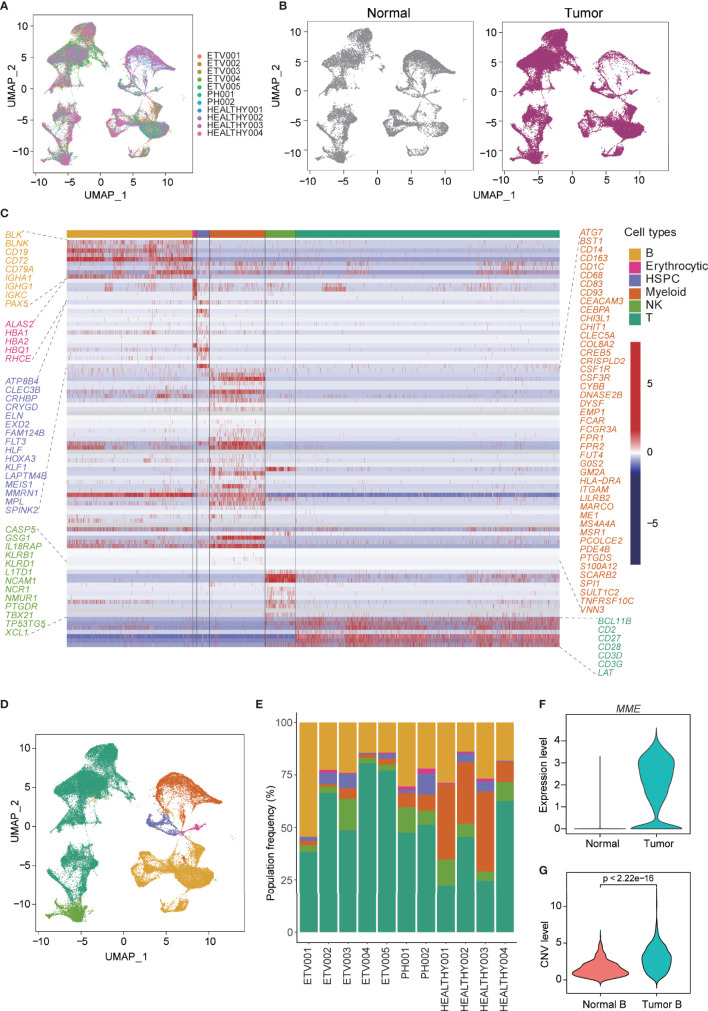
Single-cell profiling and cell-type identification in both healthy and BCP-ALL samples. **(A)** Distribution of 58,518 cells from 11 samples shown by uniform manifold approximation and projection (UMAP). **(B)** UMAP plot showing similar cell distributions in normal and tumor samples. **(C)** Gene expression heatmap of marker genes for the identification of six cell types. **(D)** UMAP visualization of six marker-based cell types. Cell types are colored as in **(C)**. **(E)** Stacked barplots showing the frequencies of six cell types in all of the 11 samples. Cell types are colored as in **(C)**. **(F)** Expression level of *MME* of B cells from normal and tumor samples. **(G)** Inferred CNV level of B cells from normal and tumor samples.

To delineate the cellular diversity of the BCP-ALL microenvironment, we analyzed the scRNA-seq data for seven newly diagnosed BCP-ALL samples (five with *ETV6*-*RUNX1* and two with *BCR*-*ABL1*, Ph+) and four healthy samples. After initial quality control was conducted (see methods), and all samples were merged using anchor-based integration, 58,518 cells (**Figure 1A**) were enrolled for downstream analyses (38,860 from BCP-ALL, 19,658 from healthy samples). Little difference was seen in the cell distribution of tumor and normal samples (**Figure 1B**). This may be due to the special sample preparation method for BCP-ALL, where 20% CD19+ B cells was mixed with 80% CD19-CD45+ non-B cells (5). The profiles separated by subtype of BCP-ALL were also very similar (**Figure S1A**). Cell-type annotation was performed using cellassign (31), and then the top genes upregulated in each cluster were examined and visualized (**Figure S1B**).All 58,518 cells were assigned to six distinct cell types: B cells (25.2%), erythrocytic cells (0.7%), hematopoietic stem and progenitor cells (HSPC 3.1%), myeloid cells (11.1%), natural killer (NK) cells (6.3%), and T cells (53.5%, **Figures 1C, D**). All 11 samples contained each of the six cell types (**Figure 1E**). After assessing the differences of non-tumor cell subsets between BCP-ALL and healthy samples, only the proportion of myeloid cells was significantly different (**Figure S1C**), which could imply a special role for myeloid in the bone marrow of BCP-ALL. According to the expression level of *MME* (an important cell surface marker in the diagnosis of human ALL), the vast majority of B cells present in neoplastic samples were leukemic cells of a pre-B phenotype (**Figures 1F**, **S1B**). The malignity of these B cells was also confirmed by inferred CNV level. Among the different cell types in the seven BCP-ALL samples, B cells had the highest CNV level (**Figure S1D**). A comparison of the CNV level of B cells between BCP-ALL and healthy samples found a significant difference (**Figure 1G**).

### Specific Ligand–Receptor Pairs Reveal an Autocrine Crosstalk Network in BCP-ALL

**Figure 2:**
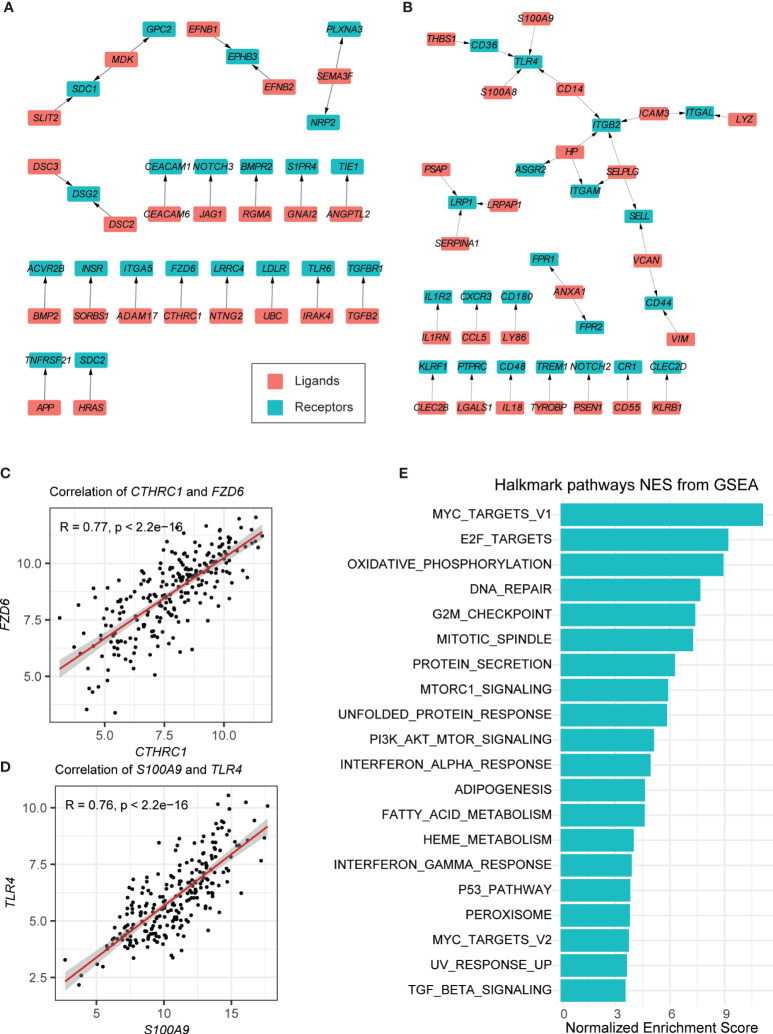
Autocrine ligand–receptor pairs network in tumor-related B cells. **(A, B)** Ligand–receptor pairs that were upregulated **(A)** and downregulated **(B)** in B cells. Red and green squares represent ligands and receptors, respectively, and arrows point from ligands to receptors. **(C, D)** Spearman correlation coefficients of two ligand–receptor pairs (*CTHRC*-*FZD6* and *S100A9-TLR4*). **(E)** GSEA of the hallmark pathways in tumor-related B cells.

The cell–cell communication level can be reflected in the expression of ligands and their special receptors. For this reason, first, we detected the intracellular communication network of B cells. Only those ligand–receptor pairs in B cells of BCP-ALL samples that had significantly high or low expression passed the filtration. We supposed that these pairs were more closely associated with leukemogenesis. As shown in **Figure S2A**, we performed differential expression testing between tumor B cells and non-tumor B cells. Then, these genes were mapped to public ligand–receptor databases (see *Materials and Methods*) (13, 27). And 152 upregulated and 206 downregulated genes were identified. Finally, the expression correlation between the individual ligands and their corresponding receptors was examined using bulk RNA-seq data obtained from our previous study (17). Only the 296 samples with *ETV6*-*RUNX1* and *BCR*-*ABL1* subtypes were used. After these strict criteria were applied, 24 upregulated and 33 downregulated ligand–receptor pairs were detected in total (see *Materials and Methods*, **Figures 2A, B**, **S3A, B****,** **Tables S1, S2**).

In the upregulated pairs, the B-cell leukemogenesis gene *FZD6* and its ligand *CTHRC1* were upregulated in several solid tumors, associated with increased cell migration and tumor invasion (34, 35). The analytical results showed that *FZD6* and *CTHRC1* were both highly expressed in the B cells of tumor samples (**Figure 2C**). It should be noted that *APP* is highly expressed in acute myeloid leukemia (AML), which may promote cancer cell proliferation and metastasis (36). In our results, we found that *APP* and its binding partner *TNFRSF21* were also highly expressed in tumor-related B-cells (**Figure S2B**). *MDK* (a cytokine and growth factor with complex biological functions involved in cancer development and progression) (37), together with its two receptors (*SDC1* and *GPC2*) were highly expressed in the B-cells of BCP-ALL samples (**Figures S2C, D**).

Among the downregulated pairs, the receptor genes *TLR4*, *ITGB2*, and *LRP1*, located in the center of the ligand–receptor network, with three to four ligands connected respectively (**Figure 2B**). This may imply that they play an important role in anti-tumorigenesis. Previous studies on these genes suggested that *TLR4* is required for protective immune response and to kill cancer cells (**Figure 2D**) (38). *ITGB2* has been found to participate in cell adhesion and cell-surface mediated signaling (39). Lower expression of *LRP1* is associated with the aggressive phenotypes and inferior clinical outcomes in some cancers (40, 41). It should be noted that *ITGAM* also has low expression in the B cells of tumor samples (**Figure S2E**). This has been reported as negative regulator of immune suppression and a target for cancer immune therapy (42).

We also conducted GSEA on B cells in BCP-ALL and healthy samples (**Figure 2E**). The enriched pathways in the HALLMARK database of neoplastic B cells were correlated with cell cycle progressions, such as the E2F targets and the G2M checkpoint, suggesting that most B cells in neoplastic samples are immature B cell progenitors. Other canonical tumor-related pathways, such as the MYC targets and the p53 pathway, were also enriched in neoplastic B cells (**Figure 2E**).

### Cell–Cell Communication From B Cells to Myeloid Cells

**Figure 3:**
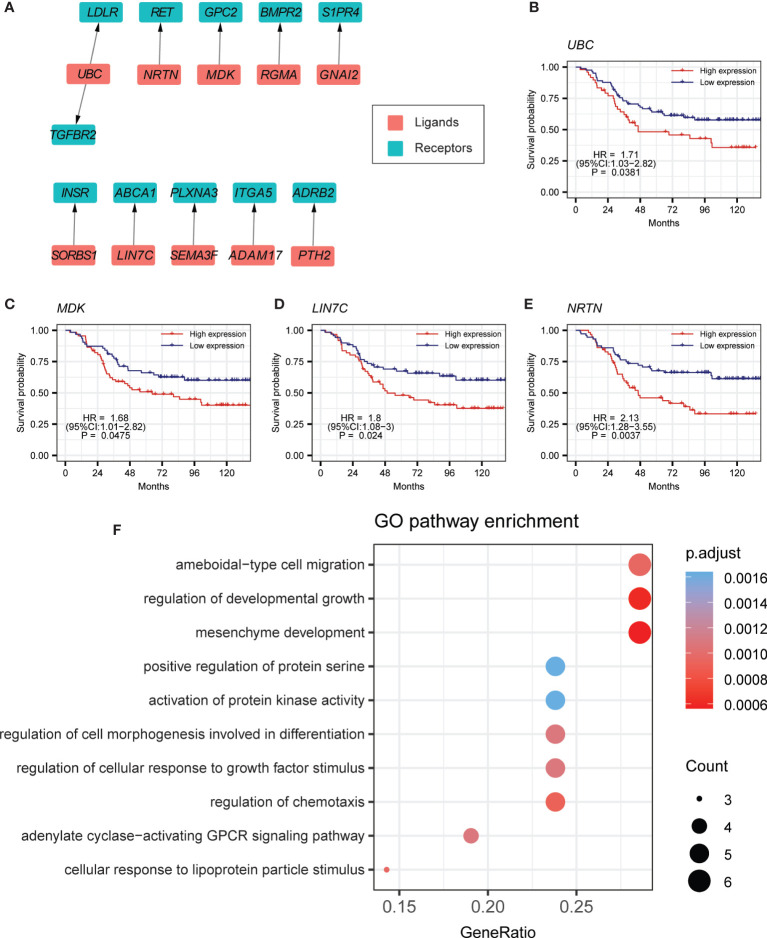
Cell–cell-communication from B cells to myeloid cells. **(A)** Ligand–receptor pairs of the signaling network from B cells to myeloid cells. Red and green squares represent ligands and receptors, respectively, and arrows point from ligands to receptors. **(B–E)** Kaplan-Meier survival for *UBC*, *MDK*, *LIN7C*, and *NRTN* in curated TARGET BCP-ALL P2 cohort. **(F)** GO pathway enrichment analysis for ligand–receptor pairs in the crosstalk from B cells to myeloid cells.

Previous studies have reported that myeloid cells might play a central role in the immune microenvironment of BCP-ALL (5, 43). Investigation of the crosstalk of B cells with myeloid cells is important for understanding the BCP-ALL TME. Thus, we performed differential expression testing between the myeloid cells of tumor samples and healthy samples. Ligands that were highly expressed in B cells and the receptors that were highly expressed in myeloid cells were selected. After calculating the Spearman’s correlation coefficient, 11 ligand–receptor pairs were identified (**Figures 3A**, **S5A, B****,** **Table S3**). Interestingly, we found that some of these 11 ligand–receptor pairs were the same as those found in the autocrine crosstalk of B cells, such as *UBC*-*LDLR* and *MDK*-*GPC2* (**Figures S4A, B**). This partly indicates the consistency in the process of leukemogenesis within the bone marrow environment. Of note, patients with higher expression level for *UBC* tend to have worse clinical outcomes (**Figure 3B**). *MDK* has similar survival trends (**Figure 3C**). The other ligand–receptor pairs that were specifically present in the crosstalk of B cells to myeloid cells, also have a crucial influence on tumorigenesis. For example, *ABCA1* is an auspicious therapy target in prostate cancer (**Figure S4C**) (44). A previous study has shown that high expression of *ADRB2* is significantly linked to early treatment failure in ALL (45) (**Figure S4D**). In the survival analyses of these specially expressed ligand–receptor pairs, patients with higher expression of *LIN7C* or *NRTN* are prone to poor prognosis (**Figures 3D, E**). Gene Ontology (GO) analysis indicated that these 11 ligand–receptor pairs are mainly associated with the biological processes of cell migration and cell development (**Figure 3F**).

### Cell–Cell Communication From Myeloid Cells to B Cells

**Figure 4:**
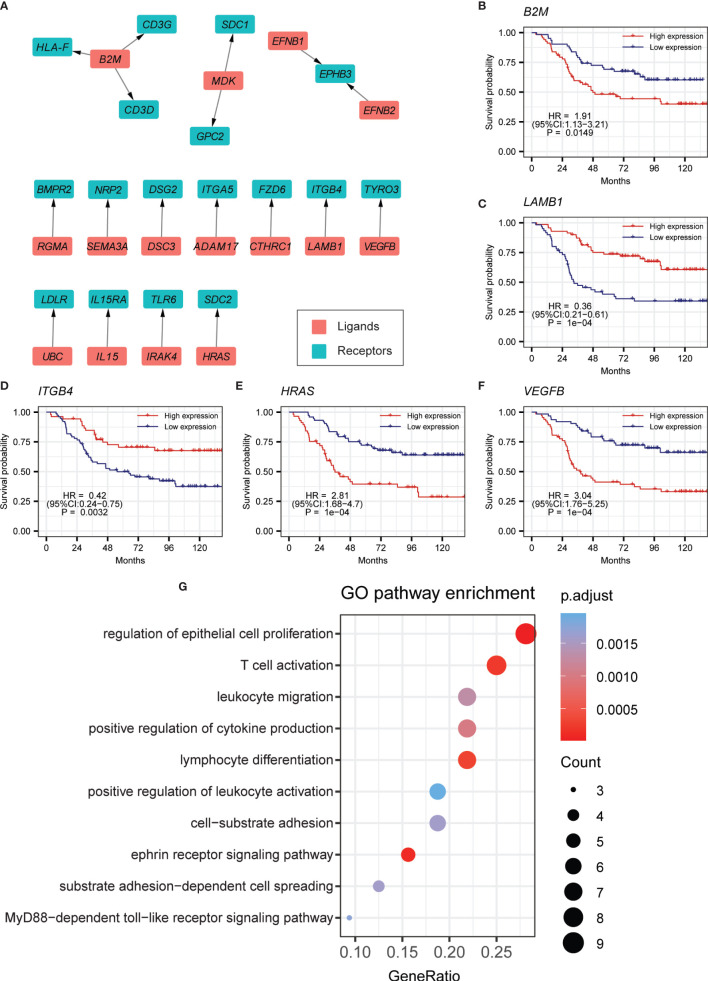
Cell–cell-communication from myeloid cells to B cells. **(A)** Ligand–receptor pairs of the signaling network from myeloid cells to B cells. Red and green squares represent ligands and receptors, respectively, and arrows point from ligands to receptors. **(B–F)** Kaplan-Meier survival for *B2M*, *LAMB1*, *ITGB4*, *HRAS*, and *VEGFB* in the curated TARGET BCP-ALL P2 cohort. **(G)** GO pathway enrichment analysis for ligand–receptor pairs in the crosstalk from myeloid cells to B cells.

We also further identified cell–cell communication from myeloid cells to B cells, built on the expression of differentially expressed ligand–receptor pairs. Ligands and receptors that were separately highly expressed in myeloid and B cells were tested. In all, 18 ligand–receptor pairs passed the strict criteria (**Figures 4A**, **S6A, B****,** **Table S4**), and about half of them match autocrine pairs of tumor-related B cells. This suggested that many interactions could be simultaneously activated by malignant or normal cells in the process of leukemogenesis. Intriguingly, the ligand *B2M* had three receptors, indicating its important role in crosstalk from myeloid cells to B cells (**Figures S4E–G**). And patients with higher expression level for *B2M* tended to have worse OS (**Figure 4B**). We also found that *LAMB1* and its receptor *ITGB4* were overexpressed in myeloid cells and B cells, respectively (**Figure S4H**). Patients with higher expression of *LAMB1* have a superior prognosis (**Figure 4C**). *ITGB4* is also a significant prognostic indicator tested by the TARGET cohort (**Figure 4D**). Besides, patients with higher expression of *HRAS* and VEGFB have worse prognoses (**Figures 4E, F**, **S4I, J**). Both of them are closely related to tumorigenesis and progression. GO analysis indicated that these ligand–receptor pairs in the crosstalk from myeloid cells to B cells were mainly related to leukocyte migration, cell proliferation, and cell activation (**Figure 4G**).

### LASSO Model Based on Ligand–Receptor Pairs Precisely Predicted BCP-ALL Patient Prognosis

The results of cell–cell communication in BCP-ALL revealed that significantly expressed ligand–receptor pairs might play a key role in leukemogenesis and progression. A machine learning model was built to predict the prognosis for BCP-ALL patients based on these pairs identified above. The principal component analysis was performed, with the expression level of ligand–receptor pairs in 14 different BCP-ALL subtypes which were classified in our previous study (17). The result showed little difference in the expression level of these ligand–receptor pairs across all the 14 BCP-ALL subgroups (**Figure S7A**).

**Figure 5:**
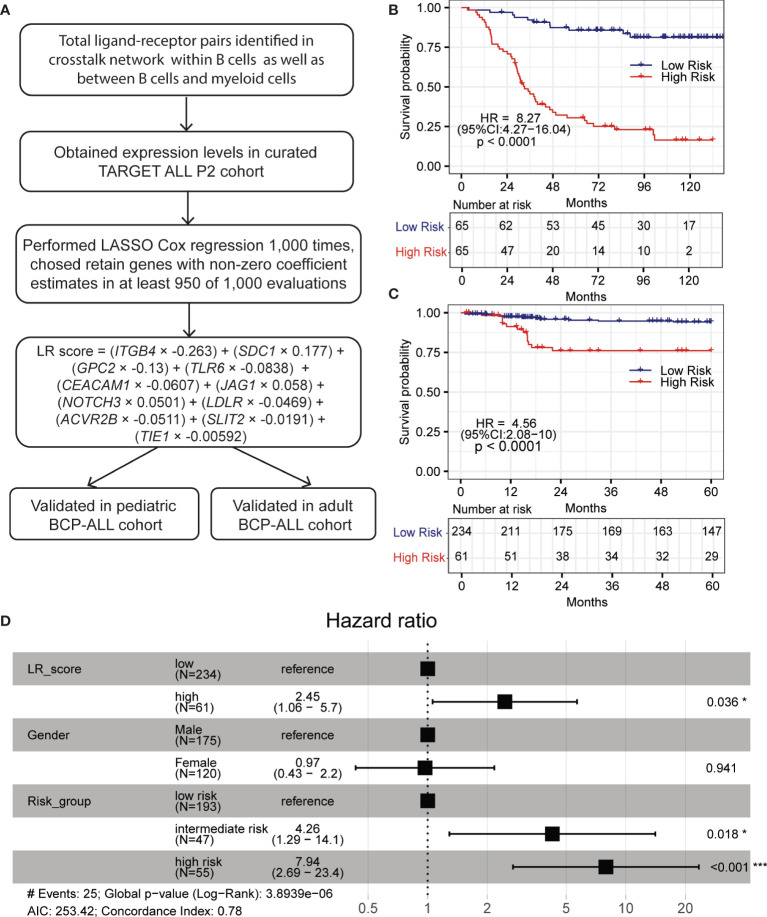
LR score based on LASSO regression model predicts inferior OS in pediatric BCP-ALL patients. **(A)** Overall scheme for constructing LASSO prognostic model. **(B, C)** High LR scores predict poor OS in the TARGET and pediatric validation cohort, respectively. **(D)** Forest plot of multivariable Cox-proportional hazard model showing LR score as an independent prognostic factor for OS in the pediatric validation cohort. Within forest plot, * indicates P-value < 0.05, *** indicates P-value < 0.001.

To develop the prognostic model, a curated TARGET cohort with 133 BCP-ALL samples was used as training cohort and samples from our previous BCP-ALL cohort were used as validation cohort (see methods). The overall process is shown in **Figure 5A** (46, 47). First, we fitted a LASSO regression model using the expression levels of ligand–receptor pairs. After performing 1,000 leave-10%-out cross-validation replications, the coefficients of 18 genes were found to be non-zero in at least one of these 1,000 evaluations (**Table S5**). And the coefficients of 11 genes were presented in at least 950 of 1,000 analyses (**Figure S7B**). Then we calculated an LR (ligand–receptor) score for each patient using the expression of these 15 genes, weighted by the regression coefficients, as defined in the LASSO model. The equation is LR score = (*ITGB4* × −0.263) + (*SDC1* × 0.177) + (*GPC2* × −0.13) + (*TLR6* × −0.0838) + (*CEACAM1* × −0.0607) + (*JAG1* × 0.058) + (*NOTCH3* × 0.0501) + (*LDLR* × −0.0469) + (*ACVR2B* × −0.0511) + (*SLIT2* × −0.0191) + (*TIE1* × −0.00592). We further used a recursive partitioning Cox regression model to dichotomize patients. After pruning the regression tree, patients in the curated TARGET cohorts with different LR scores were divided into a low-LR score group (n = 65, 50%), and a high-LR score group (n= 65, 50%). The overall survival (OS) of these two groups is remarkably different. Higher LR scores were predictive of inferior OS in TARGET cohort (HR = 8.27, 95% CI = 4.27–16.04, p < 0.0001) (**Figure 5B**).

To further confirm the robustness of the LASSO model, an independent cohort with 295 pediatric and 85 adult BCP-ALL patients was used as a validation cohort (19, 24, 25, 48). The LR score was computed with the equation defined above. A similar result was observed in pediatric patients. Based on the recursive portioning cutoff, the high-LR score group (n=61, 21%) demonstrated worse OS than the low-LR score group (n = 234, 79%), and the range of HR was 4.56 (95% CI = 2.08–10, p < 0.0001, **Figure 5C**). Although these ligand–receptor pairs were identified using scRNA-seq data of pediatric BCP-ALL patients, the prognostic power of the LR score in the 97 adult BCP-ALL patients (17) was also significant (HR = 2.99, 95% CI = 1.26–7.14, p = 0.009, **Figure S7C**). Multivariate analysis was performed with the Cox-proportional hazard model to check the individual risk factor. In the pediatric validation cohort, after adjusting for gender and risk group, the LR score remained an independent predictor of worse OS (HR = 2.45, 95% CI = 1.06–5.7, p = 0.036 **Figure 5D**). The same was true for the adult validation cohort (HR = 2.8, 95% CI = 1.18–6.8, p = 0.019, **Figure S7D**). All of these results demonstrate that the robust machine learning model built with ligand–receptor pairs has promise for identifying high-risk BCP-ALL patients and may have a role as a primary consideration for developing different treatment strategies.

## Discussion

Cellular composition and cell–cell communication are two important aspects of TME. In hematological malignant neoplasms, such as BCP-ALL, a deep understanding of cell–cell interactions in the bone marrow can help us to investigate the leukemogenesis and progression and support the development of new drugs and therapies. In the current omics studies of BCP-ALL, which mainly focus on bulk RNA-seq, the promotion of scRNA-seq reveals the landscape of TME at cellular level resolution and make it possible to investigate the cell–cell communications. In this work, we analyzed a large scale of scRNA-seq profile in seven BCP-ALL pediatric samples and four healthy samples. By classifying and identifying each cell cluster (**Figures 1D, E****)**, we found B cells from both BCP-ALL and healthy samples were mixed. This may indicate that the biological characteristics of proliferating tumor B cells were presented in a way that was similar to normal B cells. However, compared to both B cells from healthy samples and other cell types from BCP-ALL samples, tumor B cells had higher CNV level (**Figure 1G**), revealing that the accumulation of genetic abnormalities was mainly focused on B cells during leukemic progression.

T cells appeared in the largest proportion in the TME of BCP-ALL. Although previous studies of solid tumors have explored the interaction between T cells and tumor cells (49, 50), other work revealed that myeloid cells also play an important role in the TME of BCP-ALL (5). However, our understanding of the interactions involved in myeloid cells in TME remains limited. In this study, we focused on the two ways to explore the cell–cell communication: the autocrine way for B cells of tumor samples, and the paracrine way between myeloid cells and malignant B cells. Interestingly, this revealed that a considerable number of ligand–receptor pairs were closely associated with tumorigenesis and progression. For example, the *CTHRC1*-*FZD6* pair and the *APP*-*TNFRSF21* pair may significantly promote tumorigenesis and the proliferation of cancer cell (34–36). And the *LAMB1*-*ITGB4* pair has been hypothesized to be involved in tumor invasion and EMT (51). *LAMB1* has also been shown to be a potential biomarker for some cancers, such as colorectal cancer and multiple myeloma (52, 53). Pairs of *UBC*-*LDLR* and *MDK*-*GPC2* are widely overexpressed in various cell types of the BCP-ALL bone marrow microenvironment, participating in many processes of tumor development (37, 54). The ligand gene *B2M* takes center stage in the crosstalk from myeloid cells to B cells. It has been demonstrated in several studies that the elevated expression level of *B2M* is historically associated with poor outcome in several lymphoproliferative disorders, such as AML, myelodysplastic syndrome, and ALL (55). Similar results were found in our study (**Figure 4B**). Several genes in ligand–receptor pairs showed significant correlations with the clinical outcomes of pediatric BCP-ALL patients. To better predict prognosis, a machine learning model based on LASSO regression was built based on the determined ligand–receptor pairs. In the pediatric validation cohort, the prognosis for the high-LR score group was significantly worse than for the low-LR score group. Although these ligand–receptor pairs were assessed with pediatric BCP-ALL samples, our prognostic model achieved good performance in the adult validation cohort. This suggests that the prognostic model could help support the clinical decisions for both adult and pediatric BCP-ALL patients. And to further test the predictive efficiency of LR score, we applied our model in 36 tumor cohorts of TCGA. The results showed that LR score had good predictive power in a considerable number of tumors, such as acute myeloid leukemia (AML), skin cutaneous melanoma (SKCM) and uveal melanoma (UVM), of which AML was the most significant. It may indicate that LR score has strong predictive potential for prognosis in hematological malignant neoplasms (**Figure S8**).

In conclusion, *via* integrated analyses of scRNA-seq and bulk RNA-seq data for BCP-ALL, we presented a comprehensive landscape of the autocrine crosstalk network of neoplastic B cells and the paracrine communication network between B cells and myeloid cells. Based on the significant ligand–receptor pairs, a LASSO regression model was built to predict the prognoses for both pediatric and adult patients. These identifications shed light on BCP-ALL pathogenesis and have the potential to improve the clinical diagnosis for BCP-ALL patients.

## Data Availability Statement

Publicly available datasets were analyzed in this study. This data can be found here: https://www.ncbi.nlm.nih.gov/geo/query/acc.cgi?acc=GSE134759.

## Author Contributions

JH conceived, designed, and supervised the study with WZ, LW, and YD. LW collected and analyzed data, wrote the draft of the manuscript. MJ, PY, JL, WO, WZ, and CF analyzed the data and reviewed the manuscript. JH and YD oversaw the bioinformatics data analyses and modified and improved the manuscript. All authors contributed to the article and approved the submitted version.

## Funding

This work was supported by the National Natural Science Foundation of China (No. 82070147, 81570122, 81770205), the National Key Research and Development Program (No. SQ2019YFE010340), and the Shanghai Municipal Education Commission-Gaofeng Clinical Medicine Grant Support (20161303).

## Conflict of Interest

The authors declare that the research was conducted in the absence of any commercial or financial relationships that could be construed as a potential conflict of interest.
