# Supplementary material for: Single-Cell Transcriptome Analysis Identifies Ligand–Receptor Pairs Associated With BCP-ALL Prognosis
Source: Front Oncol. 2021 Mar 10;11:639013. doi: 10.3389/fonc.2021.639013 (PMC7987943; doi:10.3389/fonc.2021.639013)
Supplement: Supplementary file 1 [file DataSheet_1.pdf]

Supplementary Material

1 Supplementary Figures and Tables

1.1 Supplementary Figures

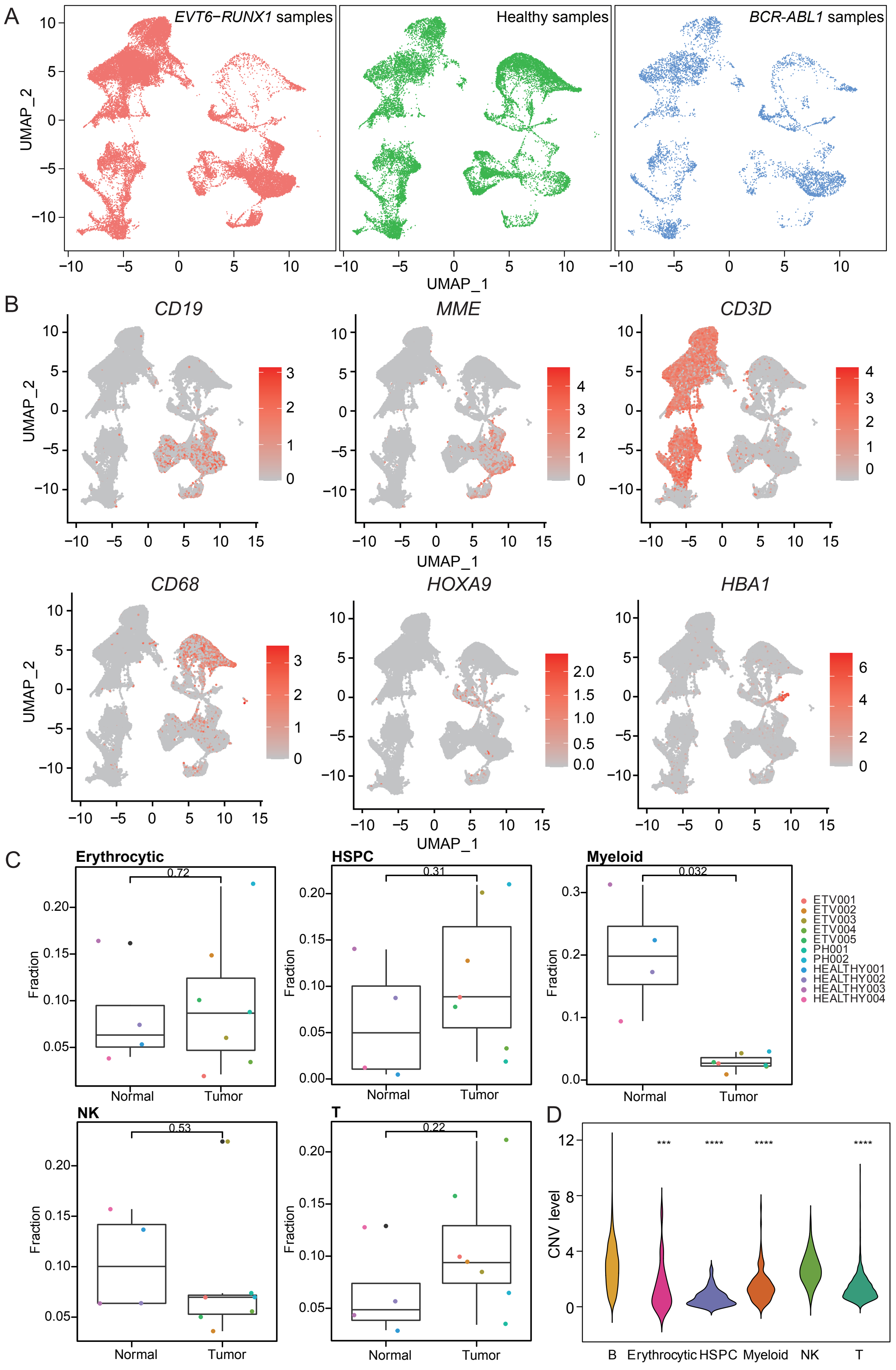
A
EVT6−RUNX1 samples
Healthy samples
BCR-ABL1 samples
UMAP_2
UMAP_1
B
CD19
MME
CD3D
CD68
HOXA9
HBA1
C
Erythrocytic
HSPC
Myeloid
NK
T
Fraction
Normal
Tumor
0.72
0.31
0.032
0.53
0.22
ETV001
ETV002
ETV003
ETV004
ETV005
PH001
PH002
HEALTHY001
HEALTHY002
HEALTHY003
HEALTHY004
D
CNV level
B
Erythrocytic
HSPC
Myeloid
NK
T

Supplementary Figure 1. Cellular heterogeneity and identification for all of 11 single-cell samples. **(A)** UMAP plot showing similar cell distributions of *ETV6-RUNX1*, healthy and *BCR-ABL1* samples. **(B)** UMAP plot showing expression levels of cell-type specific genes. **(C)** Boxplot showing fraction of non-tumor cell types of healthy and tumor samples. **(D)** Inferred CNV levels of six cell types in 7 BCP-ALL samples.

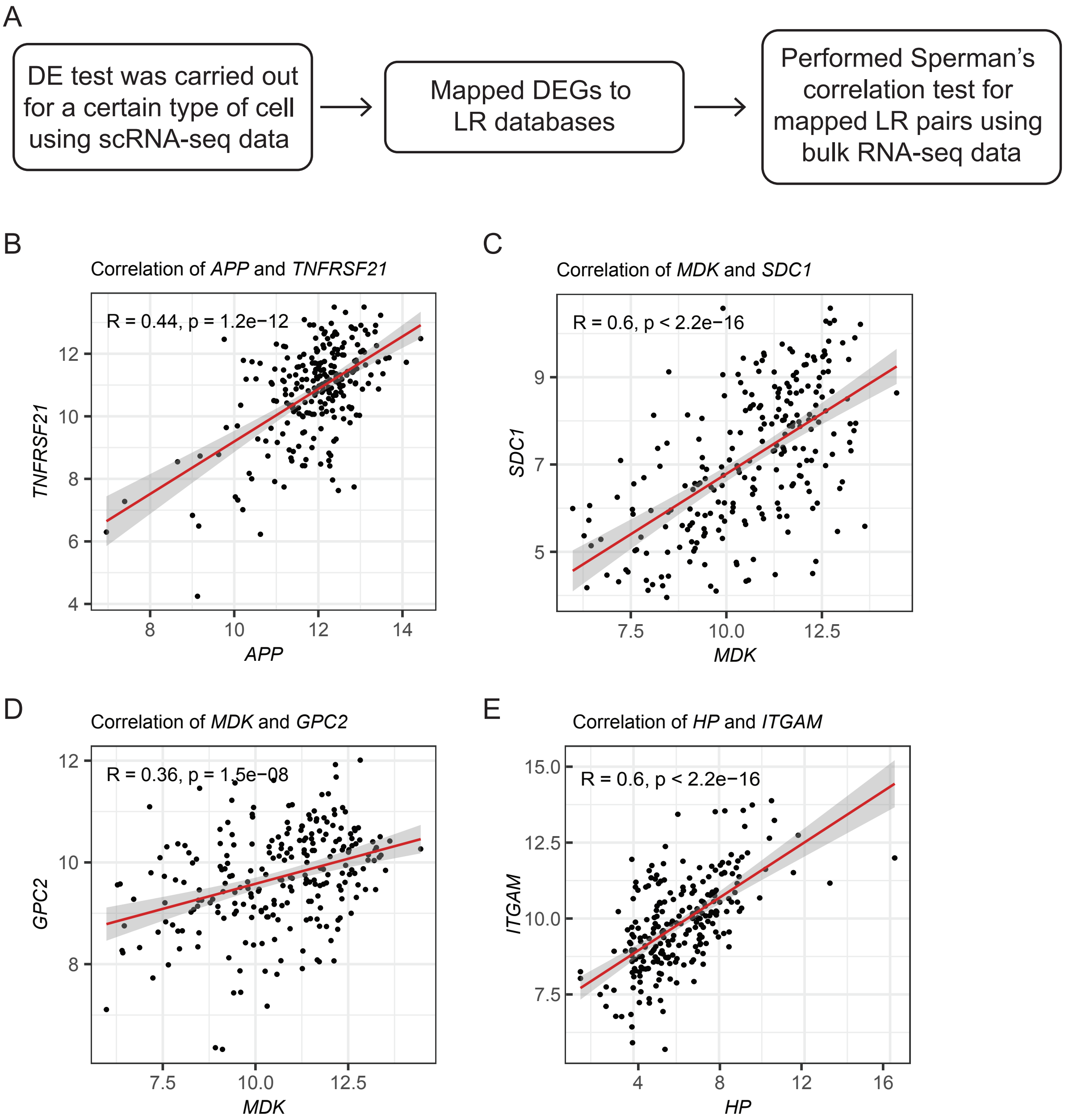

Supplementary Figure 2. Spearman correlations coefficient of ligand-receptor pairs for autocrine crosstalk network of tumor-related B cells. **(A)** Overall workflow for detecting ligand-receptor pairs. **(B-E)** Spearman correlation coefficients of four ligand-receptor pairs (*APP-TNFRSF21*, *MDK-SDC1*, *MDK-GPC2* and *HP-ITGAM*).

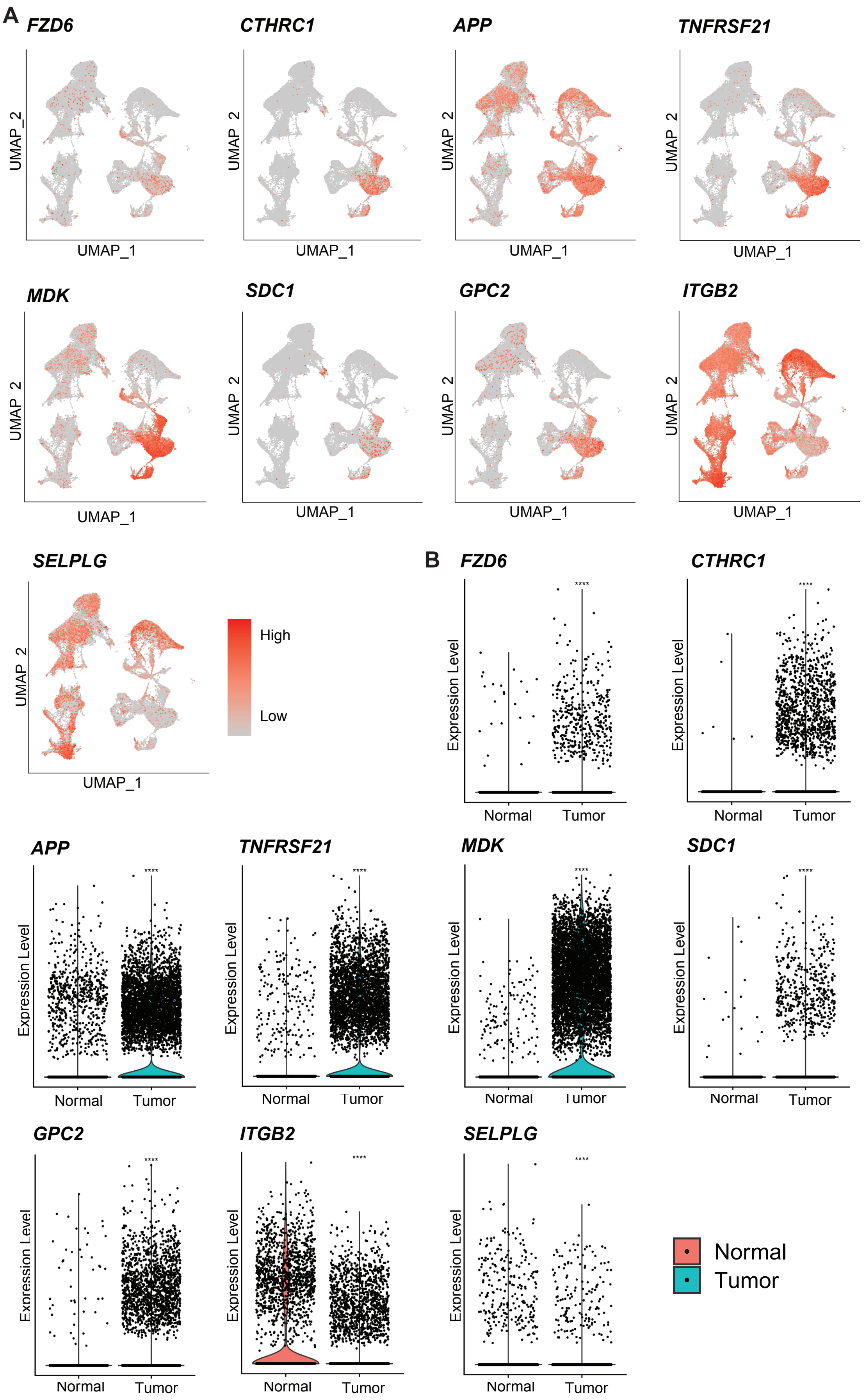

A
FZD6
CTHRC1
APP
TNFRSF21
MDK
SDC1
GPC2
ITGB2
SELPLG
UMAP_1
UMAP_2
High
Low
B
Expression Level
Normal
Tumor
****

Supplementary Figure 3. Expression levels of ligand-receptor pairs for autocrine crosstalk network of tumor-related B cells. (A) UMAP plot showing expression levels of five ligand-receptor pairs (*FZD6-CTHRC1*, *APP-TNFRSF21*, *MDK-SDC1*, *MDK-GPC2*, *ITGB2-SELPLG*). (B) Violin plot showing the differential expression of five ligand-receptor pairs between healthy and tumor samples in the corresponding cell type (*FZD6-CTHRC1*, *APP-TNFRSF21*, *MDK-SDC1*, *MDK-GPC2*, *ITGB2-SELPLG*).

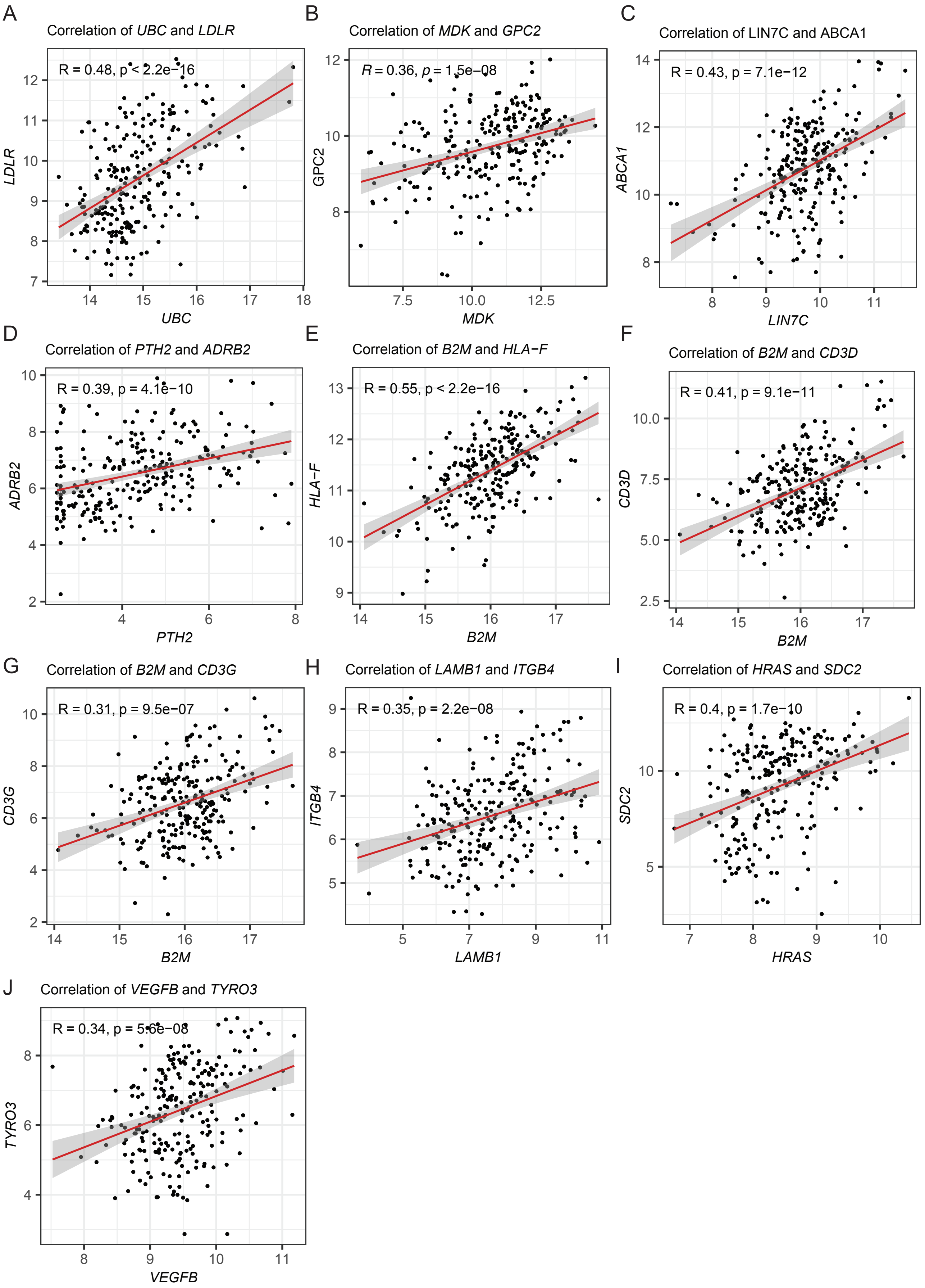
A
Correlation of UBC and LDLR
R = 0.48, p < 2.2e−16
LDLR
UBC
B
Correlation of MDK and GPC2
R = 0.36, p = 1.5e−08
GPC2
MDK
C
Correlation of LIN7C and ABCA1
R = 0.43, p = 7.1e−12
ABCA1
LIN7C
D
Correlation of PTH2 and ADRB2
R = 0.39, p = 4.1e−10
ADRB2
PTH2
E
Correlation of B2M and HLA−F
R = 0.55, p < 2.2e−16
HLA−F
B2M
F
Correlation of B2M and CD3D
R = 0.41, p = 9.1e−11
CD3D
B2M
G
Correlation of B2M and CD3G
R = 0.31, p = 9.5e−07
CD3G
B2M
H
Correlation of LAMB1 and ITGB4
R = 0.35, p = 2.2e−08
ITGB4
LAMB1
I
Correlation of HRAS and SDC2
R = 0.4, p = 1.7e−10
SDC2
HRAS
J
Correlation of VEGFB and TYRO3
R = 0.34, p = 5.6e−08
TYRO3
VEGFB

Supplementary Figure 4. Spearman correlation coefficients of ligand-receptor pairs for paracrine crosstalk network between B cells and myeloid cells. **(A-I)** Spearman correlation coefficients of nine ligand-receptor pairs (*UBC-LDLR, MDK-GPC2*, *LIN7C-ABCA1*, *PTH2-ADRB2*, *B2M-HLA-F*, *B2M-CD3D*, *B2M-CD3G*, *LAMB1-ITGB4* and *EFNB1-EPHB3*).

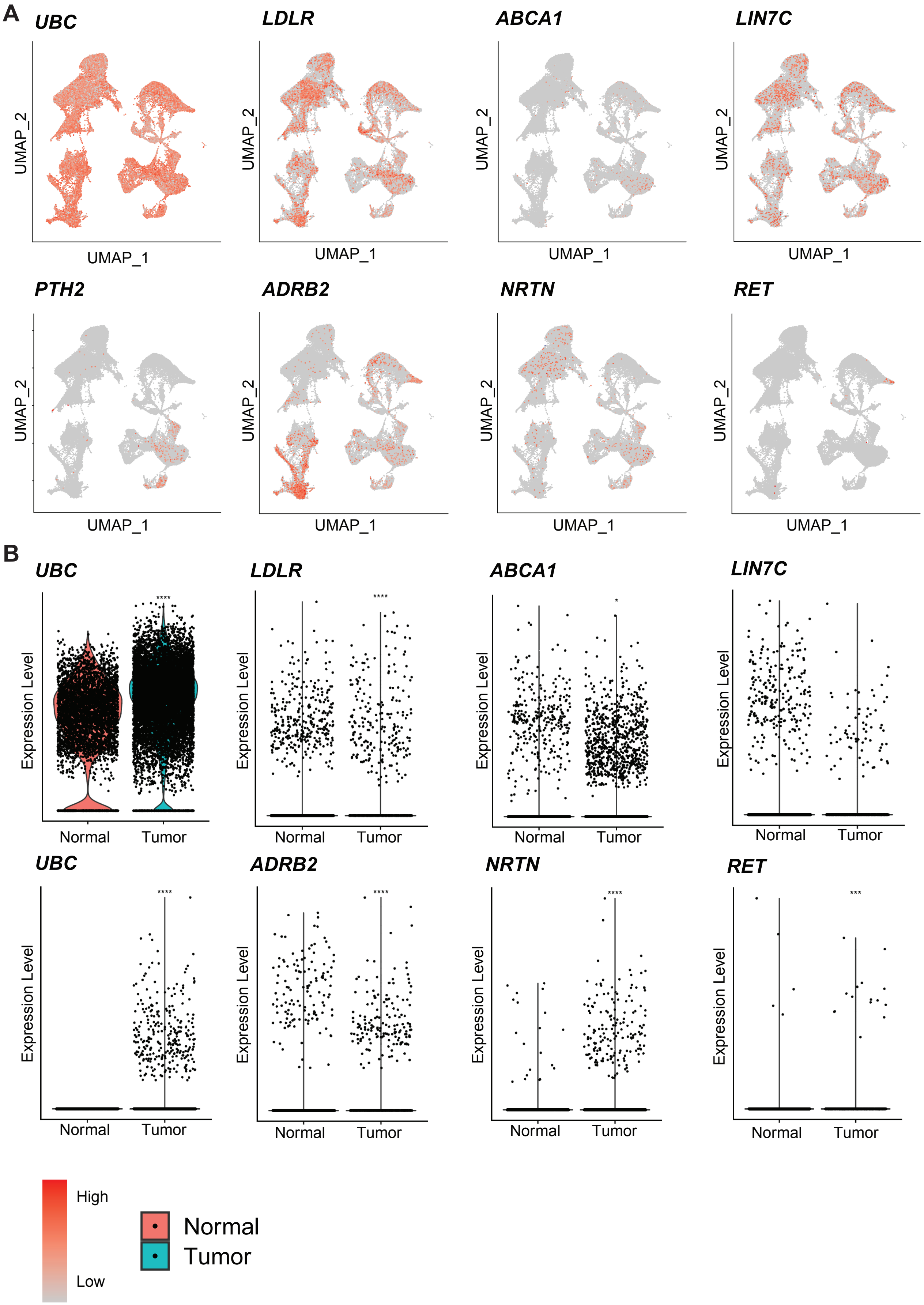
A
UBC
LDLR
ABCA1
LIN7C
PTH2
ADRB2
NRTN
RET
UMAP_1
UMAP_2
B
UBC
LDLR
ABCA1
LIN7C
UBC
ADRB2
NRTN
RET
Expression Level
Normal
Tumor
High
Low
Normal
Tumor

Supplementary Figure 5. Expression levels of ligand-receptor pairs for crosstalk from B cells to myeloid cells. (A) UMAP plot showing expression levels of four ligand-receptor pairs (*UBC-LDLR*, *ABCA1-LIN7C*, *PTH2-ADRB2*, *NRTN-RET*). (B) Violin plot showing the differential expression of four ligand-receptor pairs between healthy and tumor samples in the corresponding cell type (*UBC-LDLR*, *ABCA1-LIN7C*, *PTH2-ADRB2*, *NRTN-RET*).

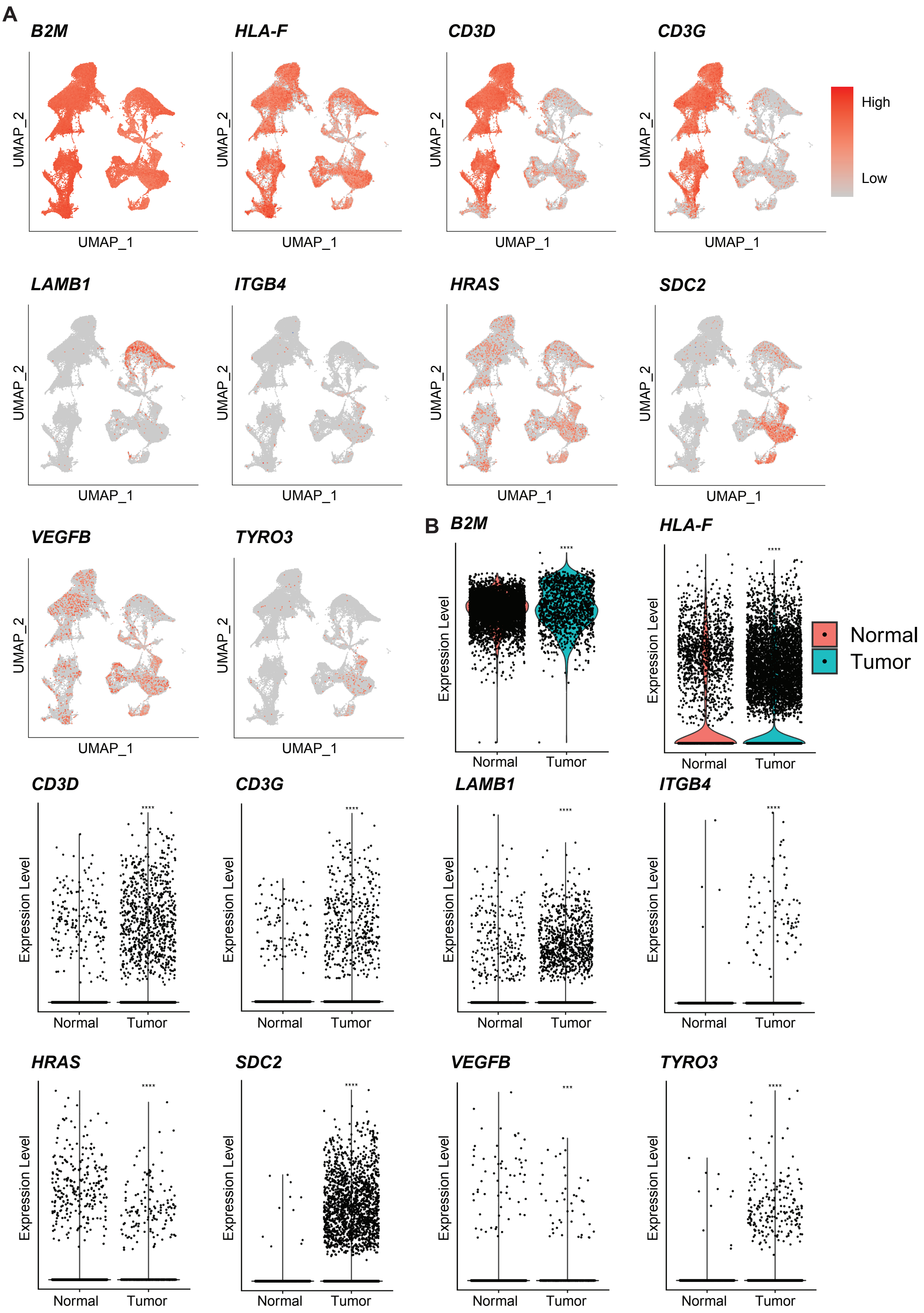
A
B2M
HLA-F
CD3D
CD3G
LAMB1
ITGB4
HRAS
SDC2
VEGFB
TYRO3
UMAP_1
UMAP_2
High
Low
B
Expression Level
Normal
Tumor

Supplementary Figure 6. Expression levels of ligand-receptor pairs for crosstalk from myeloid cells to B cells. A) UMAP plot showing expression levels of six ligand-receptor pairs (*B2M-HLA-F*, *B2M-CD3D*, *B2M-CD3G*, *LAMB1-ITGB4*, *HRAS-SDC2*, *VEGFB-TYRO3*). (B) Violin plot showing the differential expression of six ligand-receptor pairs between healthy and tumor samples in the corresponding cell type (*B2M-HLA-F*, *B2M-CD3D*, *B2M-CD3G*, *LAMB1-ITGB4*, *HRAS-SDC2*, *VEGFB-TYRO3*).

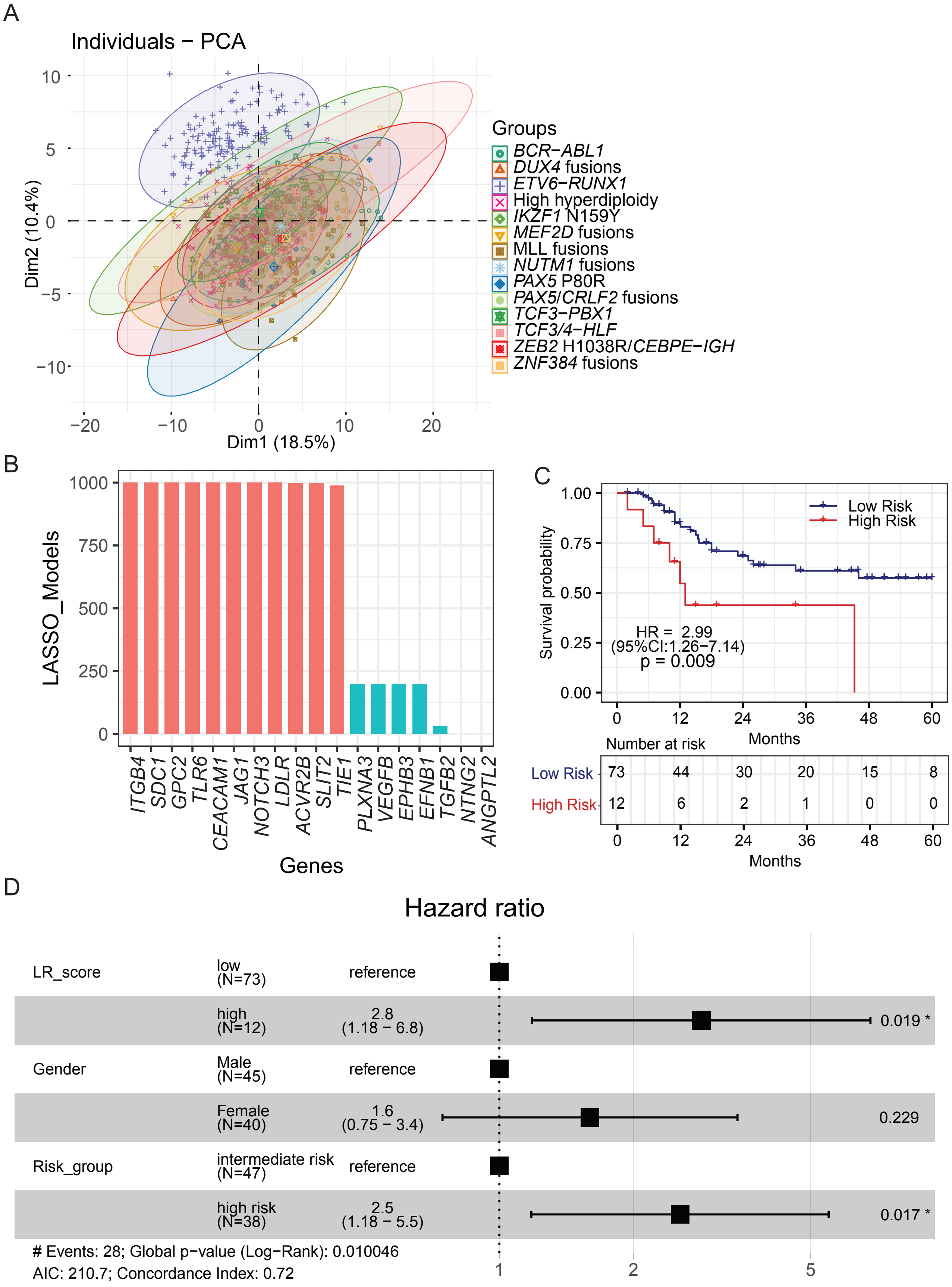
A
Individuals − PCA
Dim1 (18.5%)
Dim2 (10.4%)
Groups
BCR−ABL1
DUX4 fusions
ETV6−RUNX1
High hyperdiploidy
IKZF1 N159Y
MEF2D fusions
MLL fusions
NUTM1 fusions
PAX5 P80R
PAX5/CRLF2 fusions
TCF3−PBX1
TCF3/4−HLF
ZEB2 H1038R/CEBPE−IGH
ZNF384 fusions
B
LASSO_Models
Genes
ITGB4
SDC1
GPC2
TLR6
CEACAM1
JAG1
NOTCH3
LDLR
ACVR2B
SLIT2
TIE1
PLXNA3
VEGFB
EPHB3
EFNB1
TGFB2
NTNG2
ANGPTL2
C
Survival probability
Low Risk
High Risk
HR = 2.99
(95%CI:1.26−7.14)
p = 0.009
Months
Number at risk
Low Risk 73 44 30 20 15 8
High Risk 12 6 2 1 0 0
D
Hazard ratio
LR_score low (N=73) reference
high (N=12) 2.8 (1.18 − 6.8) 0.019 *
Gender Male (N=45) reference
Female (N=40) 1.6 (0.75 − 3.4) 0.229
Risk_group intermediate risk (N=47) reference
high risk (N=38) 2.5 (1.18 − 5.5) 0.017 *
Events: 28; Global p−value (Log−Rank): 0.010046
AIC: 210.7; Concordance Index: 0.72

Supplementary Figure 7. LR score predicted inferior OS in adult BCP-ALL patients. **(A)** PCA plot showing distribution of different BCP-ALL subgroups based on the expression of ligand-receptor pairs. **(B)** Frequency of representation of 18 genes investigated cross-validation iterations run with LASSO, 11 genes colored in red were used for developing prognostic model. **(C)** High-LR scores predicted poor OS in adult validation cohort. **(D)** Forest plot of multivariable Cox-proportional hazard model showing LR score as an independent prognostic factor for OS in the adult validation cohort.

TCGA Project	P-value	Hazard Ratio
TARGET−NBL	0.084	0.640 (0.385-1.063)
TCGA−BRCA	0.001	1.818 (1.262-2.618)
TARGET−AML	0.006	7.224 (1.775-29.40)
TARGET−WT	0.135	0.494 (0.196-1.246)
TCGA−GBM	0.109	0.720 (0.481-1.076)
TCGA−OV	0.008	0.681 (0.512-0.906)
TCGA−LUAD	<0.001	0.462 (0.340-0.627)
TCGA−UCEC	0.054	1.660 (0.992-2.776)
TCGA−KIRC	0.000	0.510 (0.376-0.691)
TCGA−HNSC	0.017	1.980 (1.129-3.472)
TCGA−LGG	0.031	0.658 (0.450-0.962)
TCGA−THCA	0.001	0.166 (0.060-0.456)
TCGA−LUSC	0.005	0.620 (0.444-0.865)
TCGA−PRAD	0.009	0.150 (0.036-0.623)
TCGA−SKCM	0.020	3.152 (1.196-8.305)
TCGA−COAD	0.002	0.516 (0.338-0.787)
TCGA−STAD	0.083	1.551 (0.945-2.547)
TCGA−BLCA	0.001	1.645 (1.218-2.222)
TCGA−LIHC	0.048	1.546 (1.004-2.378)
TCGA−CESC	0.010	1.855 (1.157-2.973)
TCGA−KIRP	0.017	2.381 (1.169-4.847)
TCGA−SARC	0.010	2.470 (1.244-4.905)
TCGA−ESCA	0.010	1.927 (1.169-3.176)
TCGA−PAAD	0.041	0.390 (0.158-0.964)
TCGA−PCPG	0.128	0.287 (0.058-1.432)
TCGA−READ	0.078	1.975 (0.927-4.210)
TCGA−TGCT	0.214	4.222 (0.436-40.87)
TCGA−THYM	0.146	2.690 (0.709-10.21)
TCGA−KICH	0.109	2.930 (0.786-10.93)
TCGA−ACC	0.091	2.498 (0.865-7.220)
TCGA−MESO	0.018	1.871 (1.114-3.141)
TCGA−UVM	0.003	6.435 (1.879-22.04)
TARGET−RT	0.072	0.412 (0.157-1.082)
TCGA−DLBC	0.110	0.322 (0.080-1.292)
TCGA−UCS	0.034	2.181 (1.061-4.482)
TCGA−CHOL	0.031	2.874 (1.103-7.486)

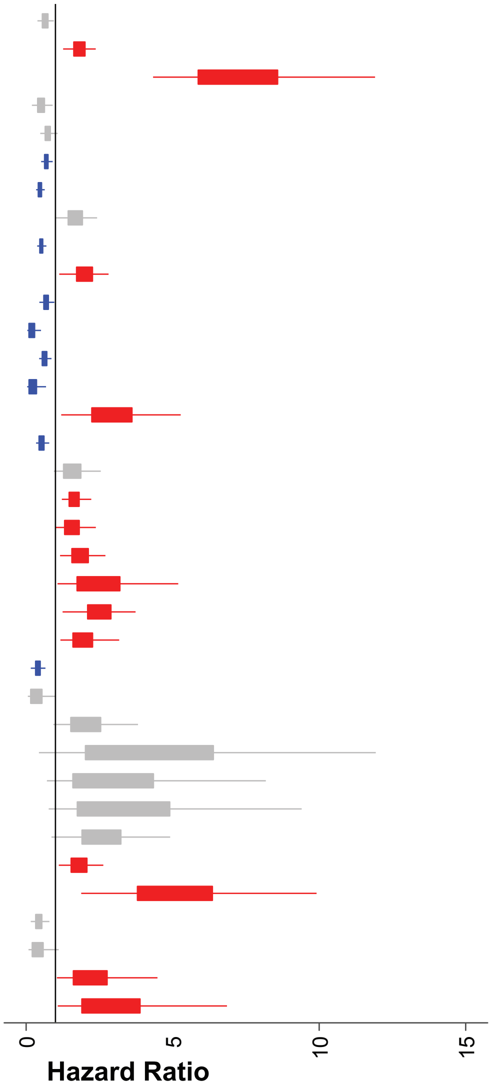

Supplementary Figure 8. Forest plot showing the predictive efficiency of LR score in 36 tumor cohort of TCGA. The tumor types marked in red represent the prognosis of high-LR score group was significantly worse than that in the low-LR score group, while those marked in blue were opposite.

1.2 Supplementary Tables

Supplementary Table 1. Ligand-receptor pairs upregulated in B cells.

Supplementary Table 2. Ligand-receptor pairs downregulated in B cells.

Supplementary Table 3. Ligand-receptor pairs from B cells to myeloid cells.

Supplementary Table 4. Ligand-receptor pairs from myeloid cells to B cells**.**

Supplementary Table 5. 18 genes which have at least once non-zero coefficient in 1000 cross-validation iterations.
